# Species of the genus *Chrysotimus* Loew from China (Diptera, Dolichopodidae)

**DOI:** 10.3897/zookeys.199.3267

**Published:** 2012-06-04

**Authors:** Mengqing Wang, Hongyin Chen, Ding Yang

**Affiliations:** 1 Key Laboratory of Integrated Pest Management in Crops, Ministry of Agriculture, Institute of Plant Protection, Chinese Academy of Agricultural Sciences, Beijing, 100081, P.R. China; 2 USDA-ARS Sino-American Biological Control Laboratory, Beijing, 100081, P.R. China; 3 Department of Entomology, Chinese Agricultural University, Beijing, 100193, China

**Keywords:** Diptera, Dolichopodidae, *Chrysotimus*, new species, China, Taxonomy

## Abstract

The following three species are described as new to science: *Chrysotimus dalongensis* **sp. n.**, *Chrysotimus huairouensis* **sp. n.**, and *Chrysotimus hubeiensis* **sp. n.**, *Chrysotimus apicicurvatus* Yang, is recorded from Palaearctic China for the first time. A key to the Chinese species of the genus is presented.

## Introduction

The genus *Chrysotimus* Loew, 1857 belongs to the subfamily Peloropeodinae. The genus is distributed worldwide except the Afrotropical region with 67 known species, 14 species are known from the Palaearctic, 25 species from the Oriental (Yang et al. 2006). Thirty-three species are known from China including those newly described herein. *Guzeriplia* Negrobov, 1968, embodies the characters of *Chrysotimus* Loew in the head and thorax with the yellow hairs and bristles and biseriate acr. Thus, it was synonymized with *Chrysotimus* by Yang et al. (2006).

## Materials and methods

Specimens were studied and illustrated with a ZEISS Stemi 2000–c stereo microscope. Genitalic preparations were made by macerating the apical portion of the abdomen in warm 10% NaOH for 17–20 min, after examination it was transferred to 75% alcohol and stored in a microvial pinned below the specimen. All specimens are deposited in the Entomological Museum of China Agricultural University (CAU), Beijing, China.

**Abbreviations are as follows:**

acracrostichal bristles


adanterodorsal bristles


avanteroventral bristles


dcdorsocentral bristles


LIfore leg


LIImid leg


LIIIhind leg


ococellar bristles


pdposterodorsal bristles


pvposteroventral bristles


vventral bristles


CuAx ratiolength of m-cu / length of distal portion of CuA.


## Systematics

### Genus Chrysotimus

Loew, 1857

http://species-id.net/wiki/Chrysotimus

Chrysotimus Loew, 1857: 48. Type-species: *Chrysotimus pusio* Loew, 1861, des. Coquillett (1910: 524).
Guzeriplia Negrobov, 1968 : 470. Type species: *Guzeriplia chlorina* Negrobov, 1968. (original designation).

#### Diagnosis.

Body with yellow or brownish hairs and bristles, small first flagellomere, most males with hind tarsomere 1 bearing several short black ventral bristles at base, and males with mid tarsomere 1 at least as long as the total of corresponding tarsomeres 2-4, male genitalia with 1-2 subepandrial processes, lateral epandrial lobe distinct.

#### Key to species (males) from China

**Table:** 

1	Hind tarsomere 1 at most with sparse black bristles at base	2
–	Hind tarsomere 1 with bundle(s) of black ventral bristles at base	10
2	Hind tarsomere 1 without black ventral bristles at base (unknown in grandis)	3
–	Hind tarsomere 1 with black ventral bristles at base	6
3	Acr present	4
–	Acr absent	5
4	Mid tarsomere 1 shorter than tarsomeres 2–5; surstylus with single lobe	*Chrysotimus beijingensis* (Yang & Saigusa)
–	Mid tarsomere 1 longer than tarsomeres 2–5; surstylus divided into 2 lobes.	*Chrysotimus grandis* Wang & Yang
5	Mid tibia with 2 pd, hind tibia with 1 ad and 2 pd; wide epandrium process nearly quadrate	*Chrysotimus guangxiensis* Yang & Saigusa
–	Mid tibia with 1 pd, hind tibia with 2 ad and 1 pd; wide epandrium process with apical concavity	*Chrysotimus sinensis* Parent
6	Antenna with 1st and 2nd antennal segments yellow	*Chrysotimus basiflavus* Yang
–	Antenna wholly black	7
7	Five dc; acr absent	*Chrysotimus apicicurvatus* Yang
–	Six dc; acr present	8
8	Nine to ten irregularly paired acr; hind tarsomere 1 with 2 black ventral bristles at base	*Chrysotimus ningxianus* Wang, Yang & Grootaert
–	Less than six irregularly paired acr; hind tarsomere 1 with 6–8 sparse black ventral bristles on basal 1/4	9
9	Hind tibia with 2 ad; epandrium with long wide and trifurcated lateral process (Figs 2–3)	*Chrysotimus dalongensis* sp. n.
–	Hind tibia with 1 ad; epandrium with short and bifurcated lateral process	*Chrysotimus acutatus* Wang, Yang & Grootaert
10	Four or five dc; acr absent	11
–	Six dc; acr present	17
11	Arista dorsal; hypandrium with broad lateral process	12
–	Arista subapical; hypandrium with thin finger-like lateral process	13
12	Hind tibia with row of v; surstylus wide	*Chrysotimus dorsalis* Yang
–	Hind tibia without row of v; surstylus slender (Figs 5–6)	*Chrysotimus huairouensis* sp. n.
13	Hind tarsomere 1 with 3–4 short black spine-like ventral bristles at base	*Chrysotimus songshanus* Wang, Yang & Grootaert
–	Hind tarsomere 1 with about 10 or more black ventral bristles at base	14
14	Hairs and bristles on thorax yellow	15
–	Hairs and bristles on thorax brownish or brown	16
15	Fore tarsomere 1 with row of about 10 v; hind tarsomere 1 with 22 short black ventral bristles on basal 1/4;surstylus basally without inner process	*chikuni* Wang, Yang & Grootaert
–	Fore tarsomere 1 without row of v; hind tarsomere 1 with less than 20 black ventral bristles on basal 1/4;surstylus basally with inner process	*Chrysotimus shennongjianus* Yang & Saigusa
16	Hind tarsomere 1 with about 12 short black ventral bristles; surstylus not furcated apically	*Chrysotimus bispinus* Yang & Saigusa
–	Hind tarsomere 1 with 15–16 short black ventral bristles; surstylus furcated apically	*Chrysotimus xuae* Wang, Yang & Grootaert
17	Acr 2–4 pairs	18
–	Acr 5 or more pairs	25
18	Hind tarsomere 1 with group of 8–12 black ventral bristles at base (which are somewhat sparse), but withoutdistinct pv	19
–	Hind tarsomere 1 with 1 (or 2) bundles of black basal ventral bristles, and row of 7–8 pv	21
19	CuAx ratio about 0.2; lateral process on epandrium not concave near middle	20
–	CuAx ratio 0.35; lateral process on epandrium concave near middle	*Chrysotimus yunlonganus* Yang & Saigusa
20	First flagellomere as long as wide; hind tibia without distinct v	*Chrysotimus lii* Wang & Yang
–	First flagellomere about 1.5 times wider than long; hind tibia with 2 pv	*Chrysotimus linzhiensis* Wang & Yang
21	R_4+5_ and M parallel apically; hind tarsomere 1 with bundle of 4–5 black ventral bristles at base	22
–	R_4+5_ and M slightly convergent apically; hind tarsomere 1 with 2 bundles of 3–4 black ventral bristles at base *Chrysotimus bifascia* Yang & Saigusa
22	Hairs and bristles on thorax yellow or pale; surstylus on epandrium not furcated apically	23
–	Hairs and bristles on thorax brown; surstylus on epandrium furcated apically	*Chrysotimus sanjiangyuanus* Wang, Yang & Grootaert
23	Fore tarsomere 1 without row of v; hind tarsomere 1 with 4–5 black ventral bristles at base	24
–	Fore tarsomere 1 with row of 5–6 v; hind tarsomere 1 with 8 black ventral bristles at base	*Chrysotimus guangdongensis* Wang, Yang & Grootaert
24	Hind femur with row of ad and pd; cercus long and narrow	*Chrysotimus xiaolongmensis* Zhang, Yang & Grootaert
–	Hind femur without distinct d; cercus round	*Chrysotimus unifascia* Yang & Saigusa
25	Hind tarsomere 1 with row of about 10 pv	26
–	Hind tarsomere 1 without distinct v	29
26	Hind tarsomere 1 with 10–12 black ventral bristles at base; mid tibia without distinct av or pv	27
–	Hind tarsomere 1 with about 20 black ventral bristles at base; mid tibia with 1 pv	28
27	First flagellomere somewhat round, 2.0 times wider than long; cercus not furcated; dorsal lobe of surstylusthick and straight	*Chrysotimus qinlingensis* Yang & Saigusa
–	First flagellomere subtriangular, about as long as wide; cercus bifurcated; dorsal lobe of surstylus thin and curved	*Chrysotimus bifurcatus* Wang & Yang
28	Fore and mid tarsomere 1 without distinct v; epandrium basally with short process	*Chrysotimus setosus* Yang & Saigusa
–	Fore and mid tarsomere 1 each with row of 5–6 v; epandrium basally with long and broad process	*Chrysotimus xiaohuangshanus* Wang, Yang & Grootaert
29	Hind tarsomere 1 with single bundle of short black ventral bristles; surstylus rather wide and thick; hypandrium with small apical incision	30
–	Hind tarsomere 1 with 2–3 bundles of 14–15 black ventral bristles on basal 1/6 (which contains 1–2 small tight bundles and 1 large loose bundle); surstylus narrow finger-like; hypandrium without apical incision	*Chrysotimus digitatus* Yang & Saigusa
30	Hairs and bristles on thorax pale or yellow; hind tarsomere 1 with 12 or less short black ventral bristles	31
–	Hairs and bristles on thorax dark brown; hind tarsomere 1 with 15–16 black ventral bristles	*Chrysotimus pingbianus* Yang & Saigusa
31	Hind tarsomere 1 with 7 or less short black ventral bristles; 6 irregularly paired acr; palpus yellow	32
–	Hind tarsomere 1 with 12 short black ventral bristles; 8 irregularly paired acr; palpus dark brown	*Chrysotimus incisus* Yang & Saigusa
32	Hind tibia with 2 pd; hind tarsomere 1 with 4–5 short black ventral bristles; surstylus slender (Fig. 8)	*Chrysotimus hubeiensis* sp. n.
–	Hind tibia with 1 pd; hind tarsomere 1 with 7–8 short black ventral bristles; surstylus very wide	*Chrysotimus lijianganus* Yang & Saigusa

### Chrysotimus dalongensis

Wang, Chen & Yang sp. n.

urn:lsid:zoobank.org:act:FC410E94-C16C-4799-96D6-AE2653EA5615

http://species-id.net/wiki/Chrysotimus_dalongensis

Figs 1–3

#### Diagnosis.

Acr 5–6 irregularly paired. Mid and hind tibiae each with 2 ad and 2 pd. Fore, mid and hind tarsomere 1 each with row of 5–7 v. Epandrium apically with long and wide lateral process, trifurcated apically; surstylus long and curved inward apically, with hook curved backwards.

#### Description.

Male. Body length 2.25–2.45 mm, wing length 2.30–2.45 mm.

**Figures 1–3.**
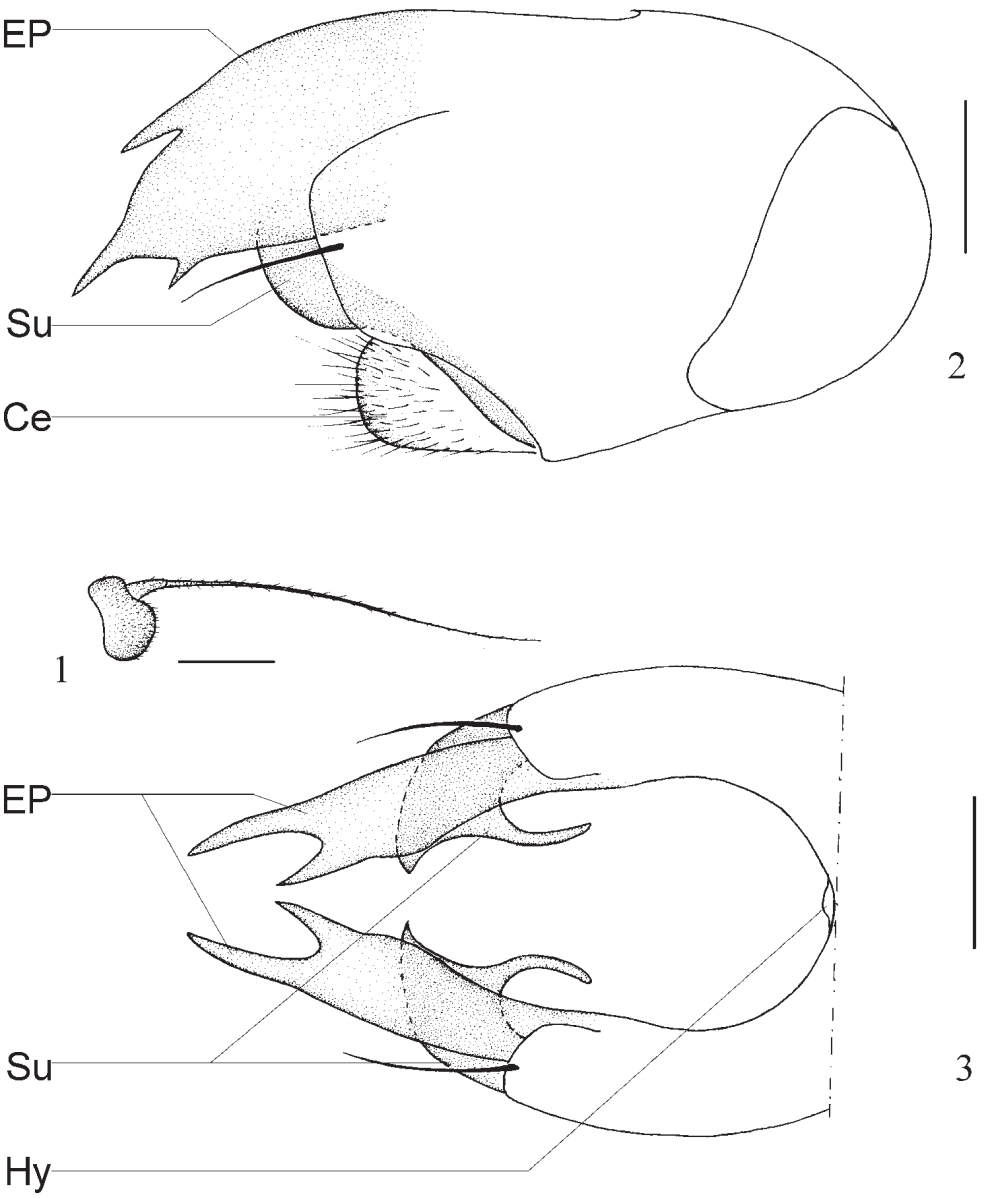
*Chrysotimus dalongensis* sp. n., male. **1** first flagellomere, lateral view **2** male genitalia, lateral view **3** tip of male genitalia, ventral view. Abbreviations: **Ce** Cerus **EP** epandrium process **Hy** hypandrium **Su** surstylus.

Head metallic green with gray pollen; frons and face brilliant. Hairs and bristles on head yellow. Ocellar tubercle weak, with 2 very long oc and 2 very short posterior hairs. Lower postocular bristles (including ventral hairs) pale. Antenna (Fig. 1) blackish; first flagellomere blackish, rather short, about 0.5 times as long as wide; arista dorsal, with basal segment very short. Proboscis brown, with brown hairs; palpus pale yellow, with pale hairs and 2 pale yellow apical bristles.

Thorax metallic green with pale gray pollen, mesonotum and scutellum brilliant. Hairs and bristles on thorax yellow; 6 dc, 5–6 irregularly paired acr short and hair-like; scutellum with 2 pairs of bristles. Propleuron with 1 pale bristle on lower portion. Legs including coxae yellow with 5th tarsomere brown. Hairs and bristles on legs yellow; coxae with yellowish hairs and bristles; fore coxa with 3–4 anterior and apical bristles, mid coxa with 2–3 anterior and apical bristles, hind coxa with 1 brown outer bristle near middle. Mid femur with 1 av apically; hind femur with 1 short av and 1 short pv apically. Mid tibia with 2 ad and 2 pd, apically with 4 bristles; hind tibia with 2 ad and 2 pd, apically with 3 bristles. Fore and mid tarsomere 1 each with row of 6–7 v. Hind tarsomere 1 with 6-8 short and thick black ventral bristles at base, and row of 5–6 pv. Relative lengths of tibia and 5 tarsomeres of legs. LI 2.4 : 1.4 : 0.6 : 0.5 : 0.3 : 0.3; LII 3.3 : 2.1 : 0.8 : 0.6 : 0.4 : 0.3; LIII 3.5 : 1.4 : 0.9 : 0.6 : 0.4 : 0.3.

Wing hyaline; veins brownish, R_4+5_ and M parallel apically; CuAx ratio 0.3. Squama yellow with pale yellow hairs. Halter pale yellow.

Abdomen metallic green with pale gray pollen, dorsum brilliant, sterna 1-2 yellow. Hairs and bristles on abdomen brown.

Male genitalia (Figs 2–3) dark brown: Epandrium distinctly longer than wide, apically with long and wide lateral process, trifurcated apically; surstylus long and curved inward apically, with curved backwards hook; cercus round, with moderately long hairs; hypandrium indistinct.

Female. Unknown.

#### Type material.

Holotype ♂, Hubei: Shennongjia, Dalongtan pound (31°75'N, 110°67'E), 30.VI.2009, Qifei Liu. Paratypes, 12♂♂, same data as holotype; 5♂♂, Hubei: Shennongjia, Dapingqian (31°75'N, 110°67'E), 7. VII. 2009, Qifei Liu. Type specimens are stored in 75% ethanol.

#### Distribution.

Known only from the type locality in Hubei.

#### Remarks.

This new species is similar to *Chrysotimus acutatus* Wang, Yang & Grootaert, but may be separated from the latter by 2 ad on hind tibia, and and by the long, wide, trifurcated lateral process on epandrium. In *Chrysotimus acutatus*, the hind tibia has 1 ad, and the lateral epandrial process is short and bifurcated (Wang et al. 2005).

#### Etymology.

The specific epithet is derived from the type locality Dalong (Hubei).

### Chrysotimus huairouensis

Wang, Chen & Yang sp. n.

urn:lsid:zoobank.org:act:8BC3F6E3-9063-499C-BB4A-A5E4EFDA77B0

http://species-id.net/wiki/Chrysotimus_huairouensis

Figs 4–6

#### Diagnosis.

First flagellomere somewhat trapeziform, about 0.8 times as long as wide. Acr absent. Hind tibia with 1 ad, 3 pd and row of v. Hind tarsomere 1 with 18–20 short and thick black ventral bristles at base. Epandrium apically with wide lateral process, process truncate apically; surstylus curved and somewhat swollen apically.

#### Description. 

Male. Body length 2.1–2.3 mm, wing length 2.0–2.3 mm.

**Figures 4–6.**
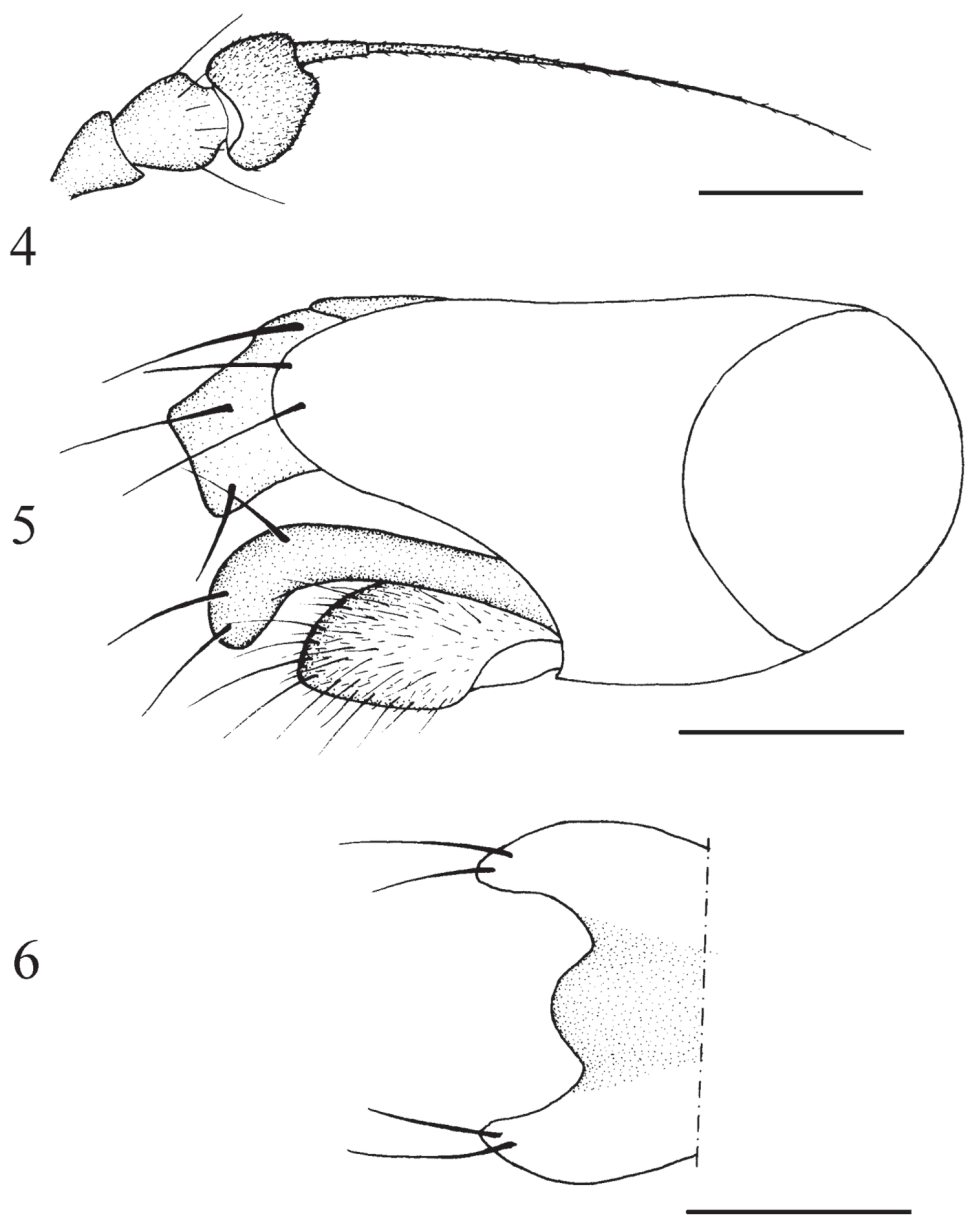
*Chrysotimus huairouensis* sp. n. **4** antenna, lateral view **5** male genitalia, lateral view **6** tip of hypandrium, ventral view.

Head metallic green with gray pollen; frons and face brilliant. Hairs and bristles on head yellow. Ocellar tubercle weak, with 2 very long oc and 2 very short posterior hairs. Lower postocular bristles (including ventral hairs) pale. Antenna (Fig. 4) blackish; first flagellomere blackish, somewhat trapeziform, rather short, about 0.8 times as long as wide; arista dorsal, with basal segment very short. Proboscis dark brown, with blackish hairs; palpus pale yellow, with pale hairs and 2 pale yellow apical bristles.

Thorax metallic green with pale gray pollen, mesonotum and scutellum brilliant. Hairs and bristles on thorax yellow; 6 dc, no acr; scutellum with 2 pairs of bristles. Propleuron with 1 pale bristle on lower portion. Legs including coxae yellow with 5th tarsomere brown. Hairs and bristles on legs brown; coxae with yellowish hairs and bristles; mid and hind coxae each with 1 brown outer bristle. Mid and hind femura each with 1 av apically. Mid tibia with 2 ad and 2 pd, apically with 4 bristles; hind tibia with 1 ad, 3 pd and one row of v, apically with 3 bristles. Hind tarsomere 1 with 18-20 short and thick black ventral bristles at base. Relative lengths of tibia and 5 tarsomeres of legs. LI 3.2 : 2.2 : 0.9 : 0.7 : 0.6 : 0.6; LII 4.2 : 2.6 : 0.9 : 0.6 : 0.4 : 0.3; LIII 5.6 : 2.4 : 1.3 : 1.0 : 0.7 : 0.6.

Winghyaline; veins brownish, R_4+5_ and M parallel apically; CuAx ratio 0.23. Squama pale yellow with yellow hairs. Halter pale yellow.

Abdomen metallic green with pale gray pollen, dorsum brilliant, sterna 1-4 yellow. Hairs and bristles on abdomen yellow.

Male genitalia (Figs 5–6) dark brown: Epandrium distinctly longer than wide, apically with wide lateral process, process truncate apically; surstylus long and finger-like, curved and somewhat swollen apically; cercus round, with moderately long hairs; hypandrium shorter than epandrium.

Female. Unknown.

#### Type material.

Holotype ♂, Beijing: Huairou, Labagou (40°32'N, 116°63'E), 29.VII.2009, Yan Li. Paratype 1 ♂, same data as holotype. Type specimens are stored in 75% ethanol.

#### Distribution.

Known only from the type locality in Beijing.

#### Remarks.

This new species is similar to *Chrysotimus dorsalis* Yang, but may be separated from the latter by the single row of v on the hind tibia, and slender surstylus. In *Chrysotimus dorsalis*, the hind tibia lacks row of v, and the surstylus is wide (Yang 2001).

#### Etymology.

The specific epithet derives from the type locality Huairou (Beijing).

### Chrysotimus hubeiensis

Wang, Chen & Yang sp. n.

urn:lsid:zoobank.org:act:A4244F92-0DE6-428A-98C9-73D0EF92DBAB

http://species-id.net/wiki/Chrysotimus_hubeiensis

Figs 7–8

#### Diagnosis.

Acr 4–5 irregularly paired. Hind tarsomere 1 with 4–5 short and thick black ventral bristles at base. Epandrium apically with short and wide lateral process, acute apically.

#### Description.

Male. Body length 2.5–2.7 mm, wing length 2.4–2.6 mm.

**Figures 7–8.**
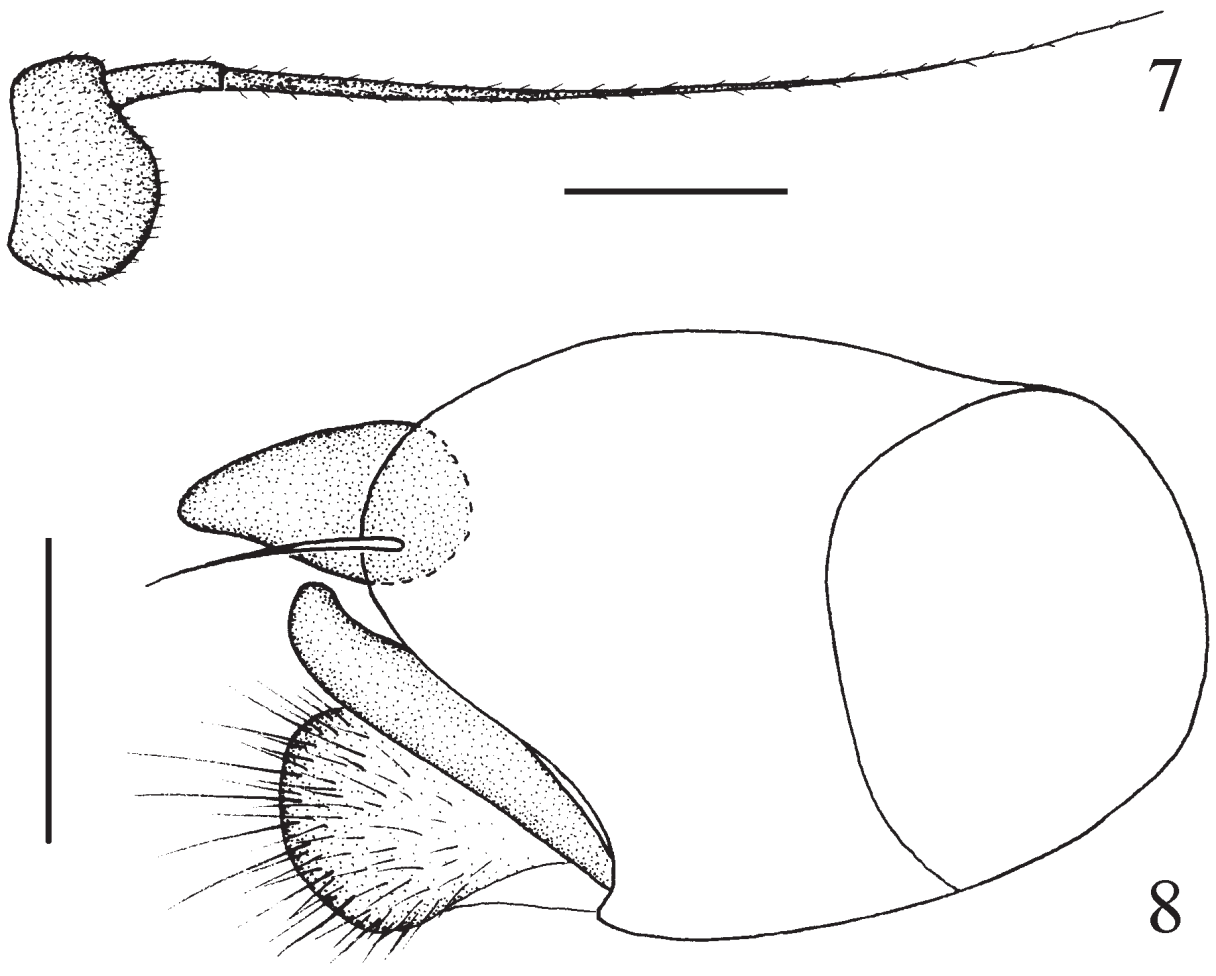
*Chrysotimus hubeiensis* sp. n. **7** first flagellomere, lateral view **8** male genitalia, lateral view.

Head metallic green with gray pollen; frons and face brilliant. Hairs and bristles on head yellow. Ocellar tubercle weak, with 2 very long oc and 2 very short posterior hairs. Lower postocular bristles (including ventral hairs) pale. Antenna blackish; first flagellomere (Fig. 7) blackish, rather short, about 0.6 times as long as wide; arista dorsal, with basal segment very short. Proboscis brown, with brown hairs; palpus pale yellow, with pale hairs and 2 pale yellow apical bristles.

Thorax metallic green with pale gray pollen, mesonotum and scutellum brilliant. Hairs and bristles on thorax yellow; 6 dc, 4–5 irregularly paired acr short and hair-like; scutellum with 2 pairs of bristles. Propleuron with 1 brown bristle on lower portion. Legs including coxae yellow with 5th tarsomeres brown. Hairs and bristles on legs dark yellow; coxae with yellowish hairs and bristles; fore coxa with 6–7 anterior and apical bristles, mid coxa with 2–3 anterior and apical bristles, hind coxa with 1 brown outer bristle near middle. Mid femur with 1 av apically; hind femur with 1 short av and 1 short pv apically. Mid tibia with 2 ad and 2 pd, apically with 3 bristles; hind tibia with 2 ad and 1 pd, apically with 3 bristles. Hind tarsomere 1 with 4-5 short and thick black ventral bristles at base. Relative lengths of tibia and 5 tarsomeres of legs. LI 4.5 : 2.3 : 1.2 : 0.9 : 0.6 : 0.7; LII 6.2 : 3.4 : 1.4 : 1.0 : 0.5 : 0.5; LIII 6.8 : 2.8 : 1.8 : 1.2 : 1.0 : 0.6.

Wing hyaline; veins brownish, R_4+5_ and M parallel apically; CuAx ratio 0.3. Squama dark yellow with brown hairs. Halter pale yellow.

Abdomen metallic green with pale gray pollen, dorsum brilliant, sterna 1-2 yellow. Hairs and bristles on abdomen brown.

Male genitalia (Fig. 8) dark brown: Epandrium distinctly longer than wide, apically with short and wide lateral process, process acute apically; surstylus slender and finger-like; cercus short and thick, with round apex.

#### Female.

Unknown.

#### Type material.

Holotype ♂, Hubei: Shennongjia, Dalongtan pound (31°75'N, 110°67'E), 1.VII.2009, Qifei Liu. Paratypes, 5♂♂, same data as holotype. Type specimens are stored in 75% ethanol.

#### Distribution.

Known only from the type locality in Hubei.

#### Remarks.

This new species is similar to *Chrysotimus lijianganus* Yang & Saigusa, but may be separated from the latter by 2 pd on hind tibia, 4-5 black ventral bristles on hind tarsomere 1, and slender, finger-like surstylus. In *Chrysotimus lijianganus*, the hind tibia has 1 pd, hind tarsomere 1 has 7–8 black ventral bristles at base, and the surstylus is very wide (Yang and Saigusa 2001b).

#### Etymology.

The specific epithet derives from the type locality Hubei.

### Chrysotimus apicicurvatus

Yang, 2001

http://species-id.net/wiki/Chrysotimus_apicicurvatus

Chrysotimus apicicurvatus Yang, 2001: 434. Type locality: China: Zhejiang, Tianmushan (Holotypes deposited in Entomological Museum of China Agricultural University, Beijng).

#### Specimens examined.

3♂♂6♀♀, Liaoning: Kuandian, Quanshan Linchang (40°73'N, 124°78'E, 650m), 9. VII. 2009, Yan Li.

#### Distribution.

Liaoning (Kuandian), Zhejiang (Tianmushan).

## Supplementary Material

XML Treatment for Chrysotimus

XML Treatment for Chrysotimus dalongensis

XML Treatment for Chrysotimus huairouensis

XML Treatment for Chrysotimus hubeiensis

XML Treatment for Chrysotimus apicicurvatus
